# Strategies for assessing the impact of loss to follow-up on estimates of neurodevelopmental impairment in a very preterm cohort at 2 years of age

**DOI:** 10.1186/s12874-021-01264-3

**Published:** 2021-06-06

**Authors:** Aurélie Piedvache, Stef van Buuren, Henrique Barros, Ana Isabel Ribeiro, Elizabeth Draper, Jennifer Zeitlin, the EPICE Research group, E. Martens, G. Martens, P. Van Reempts, K. Boerch, A. Hasselager, L. D. Huusom, O. Pryds, T. Weber, L. Toome, H. Varendi, Ile-de France, P. Y. Ancel, B. Blondel, A. Burguet, P. H. Jarreau, P. Truffert, R. F. Maier, B. Misselwitz, S. Schmidt, L. Gortner, D. Baronciani, G. Gargano, R. Agostino, D. DiLallo, F. Franco, V. Carnielli, C. Koopman-Esseboom, A. van Heijst, J. Nijman, J. Gadzinowski, J. Mazela, L. M. Graça, M. C. Machado, Carina Rodrigues, T. Rodrigues, A. K. Bonamy, M. Norman, E. Wilson E Boyle, E. S. Draper, B. N. Manktelow, A. C. Fenton, D. W. A. Milligan, J. Zeitlin, M. Bonet, A. Piedvache

**Affiliations:** 1 CRESS, Obstetrical Perinatal and Pediatric Epidemiology Research Team, EPOPé, INSERM, INRA, Université de Paris, F-75004 Paris, France; 2 Department of Methodology & Statistics, University of Utrecht, Utrecht, The Netherlands; 3 Netherlands Organisation for Applied Scientific Research TNO, Leiden, The Netherlands; 4 EPI Unit-Instituto de Saúde Pública da Universidade do Porto, Porto, Portugal; 5 University of Leicester, Leicester, UK; 6 Obstetrical, Perinatal and Pediatric Epidemiology Research Team, Center for Epidemiology and Statistics Sorbonne Paris Cité, INSERM UMR 1153, Maternité de Port-Royal, 53 avenue de l’observatoire, 75014 Paris, France

**Keywords:** Loss to follow-up, Preterm births, Neurodevelopment, Multiple imputation, Inverse probability weighting, Delta method

## Abstract

**Background:**

Loss to follow-up is a major challenge for very preterm (VPT) cohorts; attrition is associated with social disadvantage and parents with impaired children may participate less in research. We investigated the impact of loss to follow-up on the estimated prevalence of neurodevelopmental impairment in a VPT cohort using different methodological approaches.

**Methods:**

This study includes births < 32 weeks of gestational age (GA) from 4 regions in the UK and Portugal participating in a European birth cohort (*N* = 1737 survivors). Data on maternal characteristics, pregnancy complications, neonatal outcomes and neighborhood deprivation were collected at baseline. Neurodevelopment was assessed at 2 years of corrected age (CA) using standardized parent-report measures. We applied (1) multiple imputation (MI) and (2) inverse probability weighting (IPW) to estimate the impact of non-response on the prevalence of moderate to severe neurodevelopmental impairment and assessed violations of the missing at random (MAR) assumption using the delta method.

**Results:**

54.2% of children were followed-up. Follow-up was less likely when mothers were younger, multiparous, foreign-born, did not breastfeed and came from deprived areas. The prevalence of neurodevelopmental impairment was 18.4% (95% confidence interval (CI):15.9–21.1) and increased to 20.4% (95%CI: 17.3–23.4) and 20.0% (95%CI:16.9–23.1) for MI and IPW models, respectively. Simulating strong violations of MAR (children with impairments being 50% less likely to be followed-up) raised estimates to 23.6 (95%CI:20.1–27.1)

**Conclusions:**

In a VPT cohort with high loss to follow-up, correcting for attrition yielded modest increased estimates of neurodevelopmental impairment at 2 years CA; estimates were relatively robust to violations of the MAR assumption.

**Supplementary Information:**

The online version contains supplementary material available at 10.1186/s12874-021-01264-3.

## Introduction

Very preterm birth (VPT, < 32 weeks of gestation) is associated with increased infant mortality and morbidity. Survivors have higher risks of poor physical health, neurodevelopmental impairment and psychological disorders than children born at term [1–3]. Many countries have constituted longitudinal very preterm birth cohorts using population-based designs, to evaluate the longer-term health burden and to investigate the determinants of adverse long-term outcomes [4–6]. One problem facing these cohorts is loss to follow-up which varies from 25 to 50% in most cohorts, but can be up to 70% [4–7].

There are many reasons for loss to follow-up, including difficulties tracing the location of families who move, lack of time due to other family obligations or work, financial barriers and not wanting to be reminded of the circumstances of the child’s birth. Studies have shown that patients who are lost to follow-up differ from those who remain in the study, with most finding that they have lower socioeconomic status [8–11]. Some studies have also found that children who are not included are more likely to be impaired [11, 12], although having data on the full sample to investigate this bias is uncommon. Despite limited empirical evidence, there are reasons to be concerned about attrition related to the child’s health condition since the time and psychosocial consequences of raising an impaired child may make families less mobile or willing to participate in research [13]. Loss to follow-up can undermine the representativeness of estimates and introduce selection biases when the factors affecting follow-up are associated with health and developmental outcomes [14, 15].

Most studies of very preterm cohorts include a description of the characteristics of children lost to follow-up, but several analytical strategies are available for going beyond a qualitative assessment and adjusting for attrition, notably inverse probability weighting (IPW) and multiple imputation (MI) [16–19]. IPW creates a pseudo-population where individuals are weighted to represent the inverse of the probability of follow-up conditional on baseline covariates; individuals who are under-represented in the follow-up sample will be assigned a larger weight and those over-represented will have a lower weight. MI replaces each missing value with a set of plausible values based on the distribution conditional on the observed data.

These methods assume that all possible variables associated with the missingness are included in the model so that data are missing at random (MAR). Otherwise, data are missing not at random (MNAR). Checking for the MAR assumption is difficult in general because the data needed to perform such checks are missing. Sensitivity analysis can be performed in these cases. For example, we can impute under MAR, shifts to the imputations by an amount delta to account for the imperfect MAR assumption, and re-analyze the data. If the result does not change under the values for delta, then we may conclude that the estimate is robust to violations of the MAR assumption. This method is called the delta method [20, 21].

In this study, we assess how the estimated prevalence of neurodevelopmental impairment at 2 years of corrected age (CA) among very preterm infants varies under two statistical methods (IPW and MI) and study the robustness to the MAR condition.

## Methods

### Study design

This study uses data from four regions of the European Perinatal Intensive Care in Europe (EPICE) cohort. EPICE is a population-based prospective cohort of infants born before 32 weeks of gestation between March 2011 and July 2012 in 19 regions in 11 countries in Europe [22, 23]. Clinical information was collected from medical records during the neonatal hospitalization and at 2 years CA using a parental questionnaire. Four regions were selected because of the availability of small area-based socioeconomic data on children in the sample: Yorkshire & Humber and East Midlands regions from United Kingdom and Lisbon and Northern regions from Portugal.

### Study population

We included very preterm infants discharged alive from the neonatal hospitalization (*N* = 1763) and excluded children who died between discharge and 2 years (*N* = 7) and those with severe congenital anomalies (*N* = 12). For the analysis of neurodevelopment, we excluded children who were deaf or blind, because of the difficulty of assessing our primary outcome in this population (*N* = 7).

### Outcome

Moderate to severe neurodevelopmental impairment was derived from standardized questions in the parent-report questionnaire completed when the child was 2 years CA, as reported previously [24]. Briefly, this measure includes gross motor impairment based on the following questions: (1a) unable to walk without assistance or aids, (1b) unable to sit without support, (1c) unable to hold head up without support and/or (2) non-verbal cognitive (NVC) impairment based on the scale of the Parent Report of Children’s Abilities-Revised questions (PARCA-R), a parent validated screening tool [25]. The PARCA-R includes 34 items scored 0/1 from which a total NVC score is derived. Based on data from a UK term-born cohort, NVC scores < 22, corresponding to scores < 2.5th percentile were classified as moderate to severe NVC impairment [26].

### Covariables

We identified demographic and clinical factors likely to influence both the probability of follow-up and our outcome based on the literature and analyses in the EPICE cohort. These factors included maternal characteristics (age, foreign birth (Portugal)/ethnicity (UK), parity and previous cesarean section) and pregnancy and neonatal characteristics (gestational age, multiple pregnancy, small for gestational age (birthweight <10th percentile [27]), pregnancy complications (antepartum hemorrhage after 20 weeks and preterm premature rupture of membranes (PPROM), transfer in utero, presentation (breech/vertex/other), sex, Apgar score at 5 min, surfactant, respiratory support (any mechanical ventilation or nasal continuous positive airway pressure), severe neonatal morbidity (intraventricular hemorrhage grades III or IV, cystic periventricular leukomalacia, retinopathy of prematurity (grade III or more), severe necrotizing enterocolitis (defined as surgery or, peritoneal drainage)), bronchopulmonary dysplasia (defined as oxygen or respiratory support at 36 weeks post menstrual age), surgery and breastfeeding at discharge.

### Socioeconomic data

Parental socioeconomic characteristics were not collected at baseline because this information is not systematically or comparably recorded in medical records in Europe; however, information on residential postal code could be linked with small-area measures of socioeconomic deprivation in both Portugal and the UK. In Portugal, we used the European Deprivation Index built from the 2011 European Union-Statistics on Income and Living Conditions survey [28, 29]. In the UK, we used the 2015 Department for Communities and Local Government measure of deprivation, based on data collected in 2012/13 [30].

### Analysis strategy

First, we described loss to follow-up, defined as parental non-response to the two-year questionnaire for children surviving to 2 years CA. Numbers and percentages of missing values for perinatal and socioeconomic data overall and, among responders and non-responders were also reported. For responders, numbers and percentages of missing values for the outcome were also given. Then, we assessed the association of each predictor with loss to follow-up and neurodevelopmental impairment using a logistic regression adjusted for the region of birth.

The next step was to estimate the prevalence of neurodevelopmental impairment taking into consideration loss to follow-up using two different techniques. The first method was MI by chained equations [21, 31, 32]. Each missing value was replaced by 100 synthetic draws [33], and the prevalence estimates for the outcome were pooled according to Rubin’s rules. Variables that were potential predictors of missingness of the outcome as well as the outcome itself were included in MI models. Note that the MI approach imputes outcomes for children who are lost to follow-up as well as children who were followed-up, but had missing outcomes. Standard MI assumes a MAR mechanism, so we undertook a sensitivity analysis using the delta method. This consists in modifying the initial imputation model under MAR by adding a fixed quantity delta to the linear predictor before imputing data. The delta represents the difference in log-odds of having the outcome for children with missing values for the outcome compared with children with observed values. We tested a range of delta values, from 0.8 to 1.5, based on prior knowledge of the outcome to determine confidence in the prevalence estimates under the MAR assumption.

The second method for correcting for attrition was IPW where a weight was generated based on the inverse probability of follow-up [34, 35]. The probability of follow-up was estimated with a multivariate logistic regression using variables associated with follow-up. A *P*-value inferior or equal to 0.2 was used to include the possibility that some variables might be associated with the follow-up conditional on others. Missing data on predictors were imputed in a second version of the IPW method to improve the accuracy of results [36].

All calculations were performed with Stata 14.0 (Stata Corp. 2015. Stata Statistical Software: Release 14. College Station, TX: StataCorp LP). Stata coding of the delta method (see Additional file 1: Appendix for code) was informed by mice (version 3.13.0) in R [37].

## Results

At two-years, parental questionnaires were not returned for 45.8% (*n* = 796) children, varying from 32.5% in Portugal and 52.9% in the UK (Table 1). Among responders, neurodevelopmental impairment, our principal outcome, was missing for 11.1% of children meaning the prevalence of the outcome was computed on only 48.2% of children surviving at 2 years old. These missing values were mainly due to cases in UK regions (85 of the 104 missing observations). Table 1 also illustrates the impact of having missing data on perinatal variables. The complete case dataset represented 85.5% of the total sample, leading to a 14.5% loss of data with a distribution of 17.7% for non-responders and 11.7% for responders to the 2 year follow-up. The deprivation index had few missing observations (4.2%); it was well correlated with individual measures of socioeconomic disadvantage that were collected at 2 years and therefore available for the follow-up sample only (Table S1).

**Table 1:** Loss to follow-up and missing data at two years of corrected age in a very preterm cohort

		Total surviving to 2 years old^a^	Non-responders	Responders
**Total sample**		***N*** **= 1737**	***N*** **= 796 (45.8%)**	***N*** **= 941 (54.2%)**
Missing data	Perinatal and SES variablea^b,c^	251 (14.5%)	141 (17.7%)	110 (11.7%)
	Neurodevelopmental impairment at 2 years CA^b,d^	N/A	N/A	104 (11.1%)
**Portugal**		***N*** **= 603**	***N*** **= 196 (32.5%)**	***N*** **= 407 (67.5%)**
Missing data	Perinatal and SES variables^a,d^	72 (11.9%)	37 (18.9%)	35 (8.6%)
	Neurodevelopmental impairment at 2 years CA^b,d^	N/A	N/A	19 (4.7%)
**United Kingdom**		***N*** **= 1134**	***N*** **= 600 (52.9%)**	***N*** **= 534 (47.1%)**
Missing data	Perinatal and SES variables^a,d^	179 (15.8%)	104 (17.3%)	75 (14.0%)
	Neurodevelopmental impairment at 2 years CA^b,d^	N/A	N/A	85 15.9%)

^a^Death excluded; Number of deaths before age 2: total:7, Portugal:2, United Kingdom:5 ^b^SES socioeconomic status, CA corrected age ^c^see text for data items ^d^Neurodevelopmental assessment is a composite of motor disability and non-verbal cognitive disability

Table 2 shows the perinatal characteristics associated with follow-up and impairment. Neonatal morbidities and postnatal care were not associated with non-response except for children having received surfactant and those breastfeeding at discharge, who were more likely to be followed-up. In contrast, maternal factors such as younger age, having more than one child, foreign-birth/ethnicity, having had a previous cesarean section and PPROM during pregnancy were associated with non-response. Unlike the association with follow-up, neonatal morbidities and care were most strongly associated with the presence of neurodevelopmental impairment. Males were more likely to have an impairment as were children with more than three siblings. Living in the most deprived neighborhoods was strongly associated with loss to follow-up, but the association with neurodevelopmental impairment was not statistically significant. Having a missing outcome among responders was associated with being foreign-born, but not other baseline characteristics or small area deprivation (Table S2).

**Table 2:** Maternal, pregnancy and perinatal characteristics associated with loss to follow-up and the presence or absence of neurodevelopmental impairment at 2 years of corrected age (*N* = 1737)

	Responded to follow-up questionnaire			Presence of moderate to severe neurodevelopmental impairment		
	Yes (*n* = 941, 54.2%)	No (*n* = 796, 45.8%)	*p*-value	No (*n* = 683, 81.6%)	Yes (*n* = 154, 18.4%)	*p*-value
	n (%)	n (%)		n (%)	n (%)	
**Maternal characteristics**						
Maternal age						
≤ 24y	138 (14.8%)	256 (32.2%)	< 0.001	93 (13.7%)	28 (18.3%)	0.35
25y-34y	545 (58.3%)	397 (49.9%)		399 (58.7%)	85 (55.6%)	
≥ 35y	252 (27.0%)	142 (17.9%)		188 (27.6%)	40 (26.1%)	
*Missing*	*6 (0.6%)*	*1 (0.1%)*		*3 (0.4%)*	*1 (0.6%)*	
Foreign born/Ethnicity	121 (13.0%)	183 (23.2%)	< 0.001	76 (11.2%)	20 (13.2%)	0.54
*Missing*	*8 (0.9%)*	*8 (1.0%)*		*6 (0.9%)*	*2 (1.3%)*	
Multiple pregnancy	285 (30.3%)	185 (23.3%)	< 0.001	215 (31.5%)	37 (24.0%)	0.09
Parity						
First child	580 (61.8%)	367 (46.2%)	< 0.001	442 (64.8%)	86 (55.8%)	0.03
Second child	225 (24.0%)	211 (26.6%)		159 (23.3%)	37 (24.0%)	
Third or more	134 (14.3%)	216 (27.2%)		81 (11.9%)	31 (20.1%)	
*Missing*	*2 (0.2%)*	*2 (0.3%)*		*1 (0.1%)*	*0 (0.0%)*	
Previous cesarean section	90 (9.8%)	99 (13.1%)	0.03	63 (9.4%)	12 (8.1%)	0.62
*Missing*	*22 (2.3%)*	*40 (5.0%)*		*16 (2.3%)*	*5 (3.2%)*	
PPROM	203 (22.1%)	206 (27.2%)	0.01	145 (21.8%)	37 (24.3%)	0.60
*Missing*	*22 (2.3%)*	*40 (5.0%)*		*17 (2.5%)*	*2 (1.3%)*	
Antepartum hemorrhage after ≥20 weeks GA	205 (22.2%)	205 (27.0%)	0.31	139 (20.8%)	39 (25.7%)	0.19
*Missing*	*19 (2.0%)*	*36 (4.5%)*		*15 (2.2%)*	*2 (1.3%)*	
Delivery in level III	657 (70.0%)	530 (66.7%)	0.11	480 (70.5%)	110 (71.4%)	0.45
*Missing*	*2 (0.2%)*	*1 (0.1%)*		*2 (0.3%)*	*0 (0.0%)*	
Breastfeeding at discharge	649 (69.2%)	427 (53.8%)	< 0.001	486 (71.5%)	97 (63.0%)	0.04
Missing	3 (0.3%)	3 (0.4%)		3 (0.4%)	0 (0.0%)	
**Neonatal characteristics**						
Male	529 (56.2%)	426 (53.6%)	0.34	370 (54.2%)	108 (70.1%)	< 0.001
*Missing*	*0 (0.0%)*	*1 (0.1%)*		*–*	*–*	
Gestational age at birth						
23wk-25wk	70 (7.4%)	64 (8.0%)	0.49	36 (5.3%)	24 (15.6%)	< 0.001
26wk-27wk	166 (17.6%)	109 (13.7%)		119 (17.4%)	27 (17.5%)	
28wk-29wk	249 (26.5%)	228 (28.6%)		174 (25.5%)	50 (32.5%)	
30wk-31wk	456 (48.5%)	395 (49.6%)		354 (51.8%)	53 (34.4%)	
Small for gestational age^a^						
< 3th percentile	198 (21.0%)	153 (19.2%)	0.80	138 (20.2%)	42 (27.3%)	0.12
3-10th percentile	111 (11.8%)	97 (12.2%)		85 (12.4%)	18 (11.7%)	
> 10th percentile	632 (67.2%)	545 (68.6%)		460 (67.3%)	94 (61.0%)	
Apgar score < 7 (5 min)	109 (11.8%)	117 (15.0%)	0.18	67 (10.0%)	30 (19.7%)	< 0.001
*Missing*	*21 (2.2%)*	*17 (2.1%)*		*16 (2.3%)*	*2 (1.3%)*	
Surfactant	570 (60.6%)	458 (57.5%)	0.02	393 (57.5%)	111 (72.1%)	< 0.001
Any CPAP^b^	810 (86.1%)	649 (81.5%)	0.13	586 (85.8%)	136 (88.3%)	0.35
Mechanical ventilation	551 (58.6%)	456 (57.3%)	0.07	372 (54.5%)	115 (74.7%)	< 0.001
Bronchopulmonary dysplasia^c^	175 (18.8%)	156 (20.0%)	0.59	111 (16.4%)	45 (29.2%)	< 0.001
*Missing*	*9 (1.0%)*	*15 (1.9%)*		*7 (1.0%)*	*0 (0.0%)*	
Any severe morbidity	111 (11.8%)	90 (11.4%)	0.70	61 (8.9%)	38 (24.7%)	< 0.001
*Missing*	*2 (0.2%)*	*9 (1.1%)*		*0 (0.0%)*	*0 (0.0%)*	
Any congenital anomaly^d^	46 (4.9%)	40 (5.0%)	0.75	29 (4.2%)	12 (7.8%)	0.06
Any Surgery	75 (8.0%)	69 (8.7%)	0.83	39 (5.7%)	27 (17.5%)	< 0.001
Socioeconomic status						
1 (least deprivated)	236 (25.1%)	107 (13.4%)	< 0.001	182 (26.7%)	36 (23.4%)	0.54
2	193 (20.5%)	115 (14.5%)		146 (21.4%)	27 (17.5%)	
3	194 (20.6%)	144 (18.1%)		142 (20.8%)	31 (20.1%)	
4	149 (15.8%)	173 (21.7%)		102 (14.9%)	28 (18.2%)	
5 (most deprivated)	140 (14.9)	213 (26.8%)		95 (13.9%)	26 (16.9%)	
Missing	29 (3.1)	44 (5.5%)		16 (2.3%)	6 (3.9%)	

*P*-value was adjusted on region of birth and doesn’t include missing category; ^a^Using intrauterine curves; ^b^Continuous positive airway pressure; ^c^Oxygen dependency or assisted ventilation at 36 weeks post menstrual age; ^d^Congenital anomalies reported to Eurocat

Table 3 compares the prevalence of neurodevelopmental impairment before and after correction for loss to follow-up. All three approaches, IPW using the complete-case dataset, IPW using the imputed dataset, and multiple imputation, provided higher prevalence estimates, with relative increases of about 10% over the crude prevalence (range from 5.4 to 10.9%). This table also provides results separately for responders and non-responders, derived from the MI models: 18.6% (95CI (16.0%; 21.2%)) for responders versus vs 23.0% (95CI (17.4; 28.7) for non-responders.

**Table 3:** Estimated prevalence of neurodevelopmental impairment after corrections for loss to follow-up using inverse probability weighting (IPW) and multiple imputation (MI)

	Portugal	UK	Total
Crude	16.5 [13.1;20.5]	20.0 [16.6;24.0]	18.4 [15.9;21.2]
IPW complete cases	17.5 [14.4;21.1]	20.5 [17.9;23.3]	19.4 [17.4;21.6]
% Change in prevalence^a^	6.1%	2.5%	5.4%
IPW imputed cases	18.3 [14.1;22.6]	20.8 [16.7;25.0]	20.0 [16.9;23.1]
% Change in prevalence	10.9%	4.0%	8.7%
MI (total population)	17.4 [13.4;21.4]	21.9 [17.9;26.0]	20.4 [17.3;23.4]
% Change in prevalence	5.5%	9.5%	10.9%
MI (Responders)	17.1 [13.3;20.8]	19.7 [16.1;23.4]	18.6 [16.0;21.2]
% Change in prevalence	3.6%	−1.5%	1.1%
MI (Non-responders)	18.1 [8.9;27.2]	23.9 [17.2;30.6]	23.0 [17.4;28.7]

MI models were adjusted for gestational age, multiple pregnancy, foreign-born mother or foreign ethnicity, mother's age, parity, breastfeeding at discharge, previous cesarean section, PPROM, antepartum hemorrhage, sex, Apgar at 5 min less than 7, any respiratory support, surfactant, bronchopulmonary dysplasia, any severe morbidity, congenital anomaly, surgery in neonatal unit, hospitalized in a level III unit, SES status, region and follow-up

IPW model were adjusted for multiple pregnancy, foreign-born mother or foreign ethnicity, mother's age, parity, breastfeeding at discharge, previous cesarean section, PPROM, respiratory support MV and CPAP, surfactant, hospitalized in a level III unit, SES status and region

^a^percent increase in estimated prevalence corrected for selection bias compared to the crude estimate

Table 4 presents the sensitivity analyses simulating MNAR mechanism with values ranging from 0.8 to 1.5 which produced prevalence estimates from 19.0% (95% CI: 16.2 21.8) to 23.6% (95% CI: 20.1 27.1) versus 20.4% (95% CI: 17.3 23.4) under MAR. The UK regions varied from 20.5% (95% CI: 16.6 24.3) to 26.1% (95% CI: 21.3 30.8) compared to 21.9% (95% CI: 17.9 26.0), Portugal regions from 16.3% (95% CI: 12.5 20.0) to 19.0% (95% CI: 15.0 23.0) compared to 17.4% (95% CI: 13.4 21.4).

**Table 4:** Estimated prevalence of neurodevelopmental impairment under different MNAR scenarios

	delta	exponential(delta)	Portugal	UK	Total
Crude prevalence			16.5 [13.1;20.5]	20.0 [16.6;24.0]	18.4 [15.9;21.2]
MAR scenario			17.4 [13.4;21.4]	21.9 [17.9;26.0]	20.4 [17.3;23.4]
MNAR scenario	−0.2	0.8	16.3 [12.5;20.0]	20.5 [16.6;24.3]	19.0 [16.2;21.8]
	−0.1	0.9	16.6 [12.7;20.5]	21.0 [17.3;24.7]	19.5 [16.6;22.3]
	0.1	1.1	17.5 [13.5;21.5]	22.9 [18.6;27.6]	21.0 [18.0;24.1]
	0.2	1.2	18.0 [13.9;22.1]	23.7 [19.4;28.0]	21.7 [18.7;24.8]
	0.3	1.4	18.7 [14.5;22.9]	25.1 [20.5;29.7]	22.9 [19.4;26.3]
	0.4	1.5	19.0 [15.0;23.0]	26.1 [21.3;30.8]	23.6 [20.1;27.1]

## Discussion

Loss to follow-up in this sample of children born very preterm was associated with most maternal sociodemographic factors (younger age, foreign born, multiparous) as well as area-based deprivation scores, but with few clinical and neonatal variables. Using statistical methods to account for loss to follow-up led to a modest relative increase, between 5.4 and 10.9%, in the estimated prevalence of moderate to severe neurodevelopment, with marginal differences in individual country estimates. Results were consistent using MI and IPW techniques. The estimated prevalence of moderate and severe neurodevelopmental impairment was 20.4% (95% CI: 17.3–23.4) and 20.0% (95% CI: 16.9–23.1) for MI and IPW models, respectively, versus the crude prevalence of 18.4% (95% CI 15.9–21.2). Sensitivity analyses adopting the extreme assumption that the prevalence of neurodevelopment delay was 1.5 times greater for those lost to follow-up or with a missing outcome gave an estimated prevalence of 23.6% (95% CI: 20.1–27.1). The relatively small differences between the more tempered scenarios (e.g., in the range 0.8 to 1.2) suggest that the prevalence estimate is robust to small to medium violations of the MAR assumption.

In our sample, the response rate was 54.2% which is at the lower end reported in other very preterm cohorts in early childhood which range from about 50 to 90% [6, 7, 12, 38, 39, 40]. Follow-up rates tend to be higher for studies from neonatal networks when compared to population-based studies and those that have regular contact with families after discharge. Our results on the factors associated with loss to follow-up are consistent with studies on very preterm birth [5, 6, 7, 39, 40] and other cohorts [8, 9, 41, 42] which find that non–responders have lower socioeconomic status, as measured by maternal education, social deprivation scores, migrant status and young maternal age. These studies also report differences in exposures associated with socioeconomic status, such as breastfeeding and tobacco use [7, 12, 40]. These factors, in particular, maternal age, migration, maternal education and breastfeeding have all been associated with neurodevelopmental outcomes after VPT birth [43, 44].

As in our study, fewer and less consistent associations have been observed between follow-up and perinatal variables which are the strongest predictors of poor outcome for VPT children [44], as we saw in this study; many of the studies cited above report similar neonatal characteristics and morbidities for responders and non-responders. Higher birthweight has been related to lower follow-up rates in some neonatal network studies which may reflect a closer connection between families and the NICU for higher risk low-birthweight infants [38, 39]. In contrast, in some studies, families with more impaired children were less likely to attend clinical assessments or responded by postal questionnaires only versus full participation. This attrition mechanism, i.e. loss to follow-up resulting from the child’s health status, is of particular concern for VPT research.

Both IPW and MI are appropriate statistical approaches to correct estimators with baseline information and to produce standard errors that consider the uncertainty caused by missing data [16, 17, 19] and have been recommended by research advisory boards [18]. In the presence of loss to follow-up, complete-case analysis will be unbiased when data are missing completely at random or if the outcome is not included in the missingness mechanism once accounting for all remaining variables. In our analysis, these two approaches yielded similar prevalence estimates after correction, however, multiple imputation has several advantages over weighting. First, it provides an estimate of the level of impairment among non-responders conditional on fact that the imputation model was well specified. MI also allows for sensitivity analyses of the MAR assumption based on the delta method. Finally, another feature, which we could not use in this study, is the time invariance of MI, meaning that if some children are included in future follow-up waves of a cohort, their data from these time points can be used to improve precision at earlier time points.

It is difficult to compare our results to the literature because this bias is rarely quantified. In the French Epipage 2 study of very preterm births < 32 weeks of gestation, MI was used to adjust for non-response at two-years; these adjustments were generally concordant with ours: estimates of cerebral palsy prevalence rose from 4.6 to 4.8% (4% increase) and, among children without severe motor impairments, the percentage with Ages and Stages Questionnaire (ASQ) scores below the screening threshold increased from 42.0 to 47.8% (a 14% increase) [40]. The larger adjustment for the neurodevelopmental measure, compared to cerebral palsy, may reflect the stronger impact of social factors on neurodevelopment than on severe gross motor impairment [45, 46]. Corrections for attrition may also have more impact on other developmental outcomes that are more socially patterned, such as language capabilities [47] or cognition in later childhood. Having continuous as opposed to dichotomous measurements of neurodevelopment, such as parent reported scores or standardized clinical measures of cognition may also lead to different results.

Despite the importance of socioeconomic factors in determining participation in follow-up, measures of social status are often not included in baseline data because of the absence of such information in medical records. We therefore carried out our analysis in two regions which had a measure of socioeconomic status in addition to other sociodemographic or behavioral variables associated with social status which are more often available in medical records (maternal age, country of birth, parity, breastfeeding). However, although neighborhood deprivation was strongly related to follow-up it was not related to neurodevelopment in this sample.

### Strengths and limitations

Strengths of this study are the population-based design with verification of completeness at inclusion and availability of detailed perinatal data and socioeconomic deprivation based on small geographic zones. We excluded children with severe congenital anomalies or who were blind and deaf to avoid missing not at random mechanisms because these impairments may preclude parents from responding to questions not relevant to their children’s situation; Other reasons for loss to follow-up that are not observable from our data might still exist. For instance, our measure of socioeconomic status was area-based and may not fully capture social differences based on individual measures, despite good correlation with social variables in the follow-up sample. Unfortunately, among non-responders, we were not able to describe why parents did not respond to the 2 year questionnaire (explicit refusals, lack of time, postal errors, moved away etc.).

## Conclusion

Despite high attrition, adjustment for loss to follow-up in our sample led to a modest approximately 10% relative increase in estimates of neurodevelopmental impairment. Simulation exercises showed these to be relatively insensitive to MAR violations. These results are likely applicable to other cohorts, given the similarity between the factors affecting follow-up in our data and those reported in the literature. Given the wide availability of IPW and MI techniques in most software packages, systematically addressing loss to follow-up in VPT cohorts using these methods should become standard practice to provide more accurate estimates, draw attention to this important issue and build up the evidence-base on the impact of attrition in varying contexts for multiple key developmental and health outcomes.

## Supplementary Information

**Additional file 1: Table S1.** Individual socio-demographic characteristics associated with small area-based deprivation scores, sample at birth (for maternal age) and followed-up when children were 2 years of corrected age (other characteristics). **Table S2**. Baseline characteristics associated with missing neurodevelopmental impairment (*N* = 951)

## Data Availability

The datasets analysed in the current study are not publicly available because of data protection guarantees included in the ethics authorization, but are available from the corresponding author on reasonable request, in accordance with data access rules.

## Abbreviations

BPD: Bronchopulmonary Dysplasia
CA: Corrected age
CPAP: Continuous positive airway pressure
cPVL: Cystic periventricular leukomalacia (cPVL)
EPICE: European Perinatal Intensive Care in Europe
GA: Gestational age
IPW: Inverse probability weighting
IVH: Intraventricular hemorrhage (IVH)
MI: Multiple imputation
NEC: Severe necrotizing enterocolitis
NVC: Non-verbal cognition
PARCA-R: Parent Report of Children’s Abilities-Revised questions
PPROM: Preterm premature rupture of membranes
ROP: Retinopathy of prematurity
VPT: Very preterm
