# Supplementary material for: Strategies for assessing the impact of loss to follow-up on estimates of neurodevelopmental impairment in a very preterm cohort at 2 years of age
Source: BMC Med Res Methodol. 2021 Jun 6;21:118. doi: 10.1186/s12874-021-01264-3 (PMC8182922; doi:10.1186/s12874-021-01264-3)
Supplement: Supplementary file 1 — Additional file 1: Table S1. Individual socio-demographic characteristics associated with small area-based deprivation scores, sample at birth (for maternal age) and followed-up when children were 2 years of corrected age (other characteristics). Table S2. Baseline characteristics associated with missing neurodevelopmental impairment (N = 951) [file 12874_2021_1264_MOESM1_ESM.docx]

Supplementary materials

**Table S1. Individual socio-demographic characteristics associated with small area-based deprivation scores, sample at birth (for maternal age) and followed-up when children were 2 years of corrected age (other characteristics)**

|  | Deprivation score | | | | |
| --- | --- | --- | --- | --- | --- |
| Characteristics | 1 (least) | 2 | 3 | 4 | 5 (most) |
| N babies at birth (n) | 343 | 308 | 338 | 322 | 353 |
| Young mothers (≤24) (%) | 14.9 | 20.2 | 21.1 | 24.4 | 33.7 |
| N babies at 2 years (n) | 236 | 193 | 194 | 149 | 140 |
| Foreign-born (mother) (%) | 10.7 | 10.0 | 18.3 | 18.2 | 27.0 |
| Foreign-born (father) (%) | 7.0 | 10.8 | 17.2 | 20.0 | 29.0 |
| Single mother (%) | 7.3 | 10.5 | 8.3 | 21.9 | 19.9 |
| Mother is unemployed (%) | 10.6 | 9.5 | 12.5 | 19.9 | 24.5 |
| Father is unemployed (%) | 3.2 | 3.6 | 5.7 | 10.3 | 13.5 |
| Mother has low educational level^1^ (%) | 26.5 | 33.0 | 51.1 | 61.4 | 64.3 |
| Father has low educational level^1^ (%) | 43.1 | 49.7 | 59.1 | 68.2 | 75.2 |

(1) Low education: a level of education equivalent to upper secondary education or higher

**Table S2. Baseline characteristics associated with missing neurodevelopmental impairment (N=951)**

|  | Non-Missing (N=837) | Missing  (N=104) |  |
| --- | --- | --- | --- |
| Characteristics | n (%) | n (%) | p-value |
| Maternal characteristics |  |  |  |
| Maternal age (years) |  |  |  |
| ≤24 | 121 (14.5%) | 17 (16.7%) | 0.82 |
| 25-34 | 484 (58.1%) | 61 (59.8%) |  |
| ≥35 | 228 (27.4%) | 24 (23.5%) |  |
| Missing | *4 (0.5%)* | *2 (1.9%)* |  |
| Foreign born/Ethnicity | 96 (11.6%) | 25 (24.0%) | 0.00 |
| *Missing* | 8 (1.0%) | 0 (0.0%) |  |
| Parity |  |  |  |
| First child | 528 (63.2%) | 52 (50.5%) | 0.11 |
| Second | 196 (23.4%) | 29 (28.2%) |  |
| Third or more | 112 (13.4%) | 22 (21.4%) |  |
| Missing | 1 (0.1%) | 1 (1.0%) |  |
| Multiple pregnancy | 252 (30.1%) | 33 (31.7%) | 0.63 |
| Previous cesarean section | 75 (9.2%) | 15 (14.6%) | 0.08 |
| *Missing* | *21 (2.5%)* | *1 (1.0%)* |  |
| Any antepartum hemorrhage after ≥ 20 weeks of gestation | 178 (21.7%) | 27 (26.5%) | 0.83 |
| *Missing* | *17 (2.0%)* | *2 (1.9%)* |  |
| Preterm contractions | 402 (48.9%) | 53 (52.0%) | 0.77 |
| *Missing* | *15 (1.8%)* | *2 (1.9%)* |  |
| Infection | 48 (5.7%) | 8 (7.7%) | 0.23 |
| *Missing* | *2 (0.2%)* | *0 (0.0%)* |  |
| Diagnosis of IUGR | 120 (14.7%) | 11 (10.8%) | 0.74 |
| *Missing* | *21 (2.5%)* | *2 (1.9%)* |  |
| PPROM | 182 (22.2%) | 21 (20.8%) | 0.70 |
| *Missing* | *19 (2.3%)* | *3 (2.9%)* |  |
| Preeclampsia/eclampsia/HELLP^1^ | 144 (17.5%) | 16 (15.7%) | 0.93 |
| *Missing* | *16 (1.9%)* | *2 (1.9%)* |  |
| Cesearean section | 538 (64.4%) | 64 (61.5%) | 0.87 |
| *Missing* | *1 (0.1%)* | *0 (0.0%)* |  |
| Received ANS | 758 (91.3%) | 95 (91.3%) | 0.86 |
| *Missing* | *7 (0.8%)* | *0 (0.0%)* |  |
| Delivery in level III | 590 (70.7%) | 67 (64.4%) | 0.38 |
| *Missing* | *2 (0.2%)* | *0 (0.0%)* |  |
| Male | 478 (57.1%) | 51 (49.0%) | 0.17 |
| Breastfeeding at discharge | 583 (69.9%) | 66 (63.5%) | 0.22 |
| *Missing* | *3 (0.4%)* | *0 (0.0%)* |  |

| Neonatal characteristics |  |  |  |
| --- | --- | --- | --- |
| Gestational age at birth (weeks) |  |  |  |
| 23-25 | 60 (7.2%) | 10 (9.6%) | 0.66 |
| 26-27 | 146 (17.4%) | 20 (19.2%) |  |
| 28-29 | 224 (26.8%) | 25 (24.0%) |  |
| 30-31 | 407 (48.6%) | 49 (47.1%) |  |
| Small for gestational age |  |  |  |
| <3th percentile | 180 (21.5%) | 18 (17.3%) | 0.29 |
| 3-10th percentile | 103 (12.3%) | 8 (7.7%) |  |
| > 10th percentile | 554 (66.2%) | 78 (75.0%) |  |
| Apgar score <7 at 5 minutes | 97 (11.8%) | 12 (11.9%) | 0.63 |
| *Missing* | *18 (2.2%)* | *3 (2.9%)* |  |
| Inborn | 745 (89.0%) | 90 (86.5%) | 0.82 |
| At least one transfer in neonatal care | 286 (34.2%) | 45 (43.3%) | 0.67 |
| Mechanical ventilation | 487 (58.2%) | 64 (61.5%) | 0.88 |
| Any CPAP | 722 (86.3%) | 88 (84.6%) | 0.86 |
| Surfactant | 504 (60.2%) | 66 (63.5%) | 0.86 |
| Any Surgery | 66 (7.9%) | 9 (8.7%) | 1.00 |
| Any reportable congenital anomaly | 41 (4.9%) | 5 (4.8%) | 0.97 |
| Bronchopulmonary dysplasia | 156 (18.8%) | 19 (18.6%) | 0.39 |
| *Missing* | *7 (0.8%)* | *2 (1.9%)* |  |
| Any severe morbidity | 99 (11.8%) | 12 (11.8%) | 0.82 |
| *Missing* | *0 (0.0%)* | *2 (1.9%)* |  |
| Deprivation Index |  |  |  |
| 1 | **256 (31.3%)** | **23 (23.7%)** | **0.36** |
| 2 | 178 (21.8%) | 20 (20.6%) |  |
| 3 | **142 (17.4%)** | **22 (22.7%)** |  |
| 4 | 130 (15.9%) | 20 (20.6%) |  |
| 5 | **112 (13.7%)** | **12 (12.4%)** |  |
| Missing | 19 (2.3%) | 7 (6.7%) |  |

(1) HELLP: Haemolysis, Elevated Liver enzyme levels, and Low Platelet levels

## Stata program for the delta method.

local i=0

forv k=-0.2(0.1)0.4 {

use "mydataset", clear

local i = `i' + 1

*generate a delta only for outcome missing

gen delta = 0 if outcome != .

replace delta = `k' if outcome == .

mi set wide

mi register imputed outcome covariates_missing

mi register regular covariates_nonmissing

mi impute chained ///

(logit, offset(delta)) outcome ///

(logit) covariates_missing ///

= i.(covariates_nonmissing) ///

, add(100) noisily augment force rseed(123456789)

save "mydataset_delta_`i'", replace

}

## R program for the delta method.

library("mice")

library("mitools")

delta <- c("-0.2","-0.1","0","0.1","0.2","0.3","0.4")

imp.mnar.delta <- vector("list", length(delta))

init <- mice(mydataset, maxit = 0)

meth = init$method

predM = init$predictorMatrix

meth["outcome"] <-"mnar.logreg"

for (i in 1:length(delta)) {

mnar.blot <- list(outcome = list(ums = delta[i]))

imp.mnar.delta[[i]] = mice(mydataset, pred=predM, method = meth, seed=123,

blots = mnar.blot, m=100, maxit=10)

}
